# Improved Small Object Detection Algorithm CRL-YOLOv5

**DOI:** 10.3390/s24196437

**Published:** 2024-10-04

**Authors:** Zhiyuan Wang, Shujun Men, Yuntian Bai, Yutong Yuan, Jiamin Wang, Kanglei Wang, Lei Zhang

**Affiliations:** 1 School of Information Science and Engineering, Yanshan University, Qinhuangdao 066004, China; zhiyuanwang@stumail.ysu.edu.cn (Z.W.); menshujun@stumail.ysu.edu.cn (S.M.); ysuyyt@stumail.ysu.edu.cn (Y.Y.); wjm@stumail.ysu.edu.cn (J.W.); wangkl@stumail.ysu.edu.cn (K.W.); 2 Silesian College of Intelligent Science and Engineering, Yanshan University, Qinhuangdao 066004, China; baiyuntian186@stumail.ysu.edu.cn

**Keywords:** small object detection, attention mechanisms, contextual information, YOLOv5, digital images, spatial resolution

## Abstract

Detecting small objects in images poses significant challenges due to their limited pixel representation and the difficulty in extracting sufficient features, often leading to missed or false detections. To address these challenges and enhance detection accuracy, this paper presents an improved small object detection algorithm, CRL-YOLOv5. The proposed approach integrates the Convolutional Block Attention Module (CBAM) attention mechanism into the C3 module of the backbone network, which enhances the localization accuracy of small objects. Additionally, the Receptive Field Block (RFB) module is introduced to expand the model’s receptive field, thereby fully leveraging contextual information. Furthermore, the network architecture is restructured to include an additional detection layer specifically for small objects, allowing for deeper feature extraction from shallow layers. When tested on the VisDrone2019 small object dataset, CRL-YOLOv5 achieved an mAP50 of 39.2%, representing a 5.4% improvement over the original YOLOv5, effectively boosting the detection precision for small objects in images.

## 1. Introduction

Small object detection is vital in various applications, including remote sensing image analysis, medical image diagnostics, and intelligent traffic systems. Rapid and accurate detection of small objects, such as unauthorized ships or aircraft in remote sensing images, can significantly enhance national defense security. In the medical field, precise detection of small lesions, like lung nodules, retinal abnormalities, or early-stage tumors, is crucial for early diagnosis and treatment of diseases. Similarly, in intelligent traffic systems, the swift and accurate identification of pedestrians, vehicles, or traffic signs plays a key role in maintaining traffic order and enhancing safety. However, detecting small objects in images is inherently challenging due to factors like limited pixel counts, difficulty in feature extraction, and complex backgrounds. As a result, the task of fast and accurate small object detection has become a focal point of research and innovation in the scientific community in recent years.

Traditional methods for small object detection are often hindered by complex background interference, which adversely affects feature extraction. Deep learning-based object detection algorithms exhibit strong feature extraction capabilities and robustness [1]. These algorithms are broadly categorized into single-stage and two-stage approaches. Two-stage methods, exemplified by R-CNN [2], Fast R-CNN [3], Faster R-CNN [4], and Mask R-CNN [5], typically require initial region proposal generation followed by feature extraction and classification, making them time-consuming compared to single-stage methods. Single-stage detectors like the YOLO [6,7,8] series and SSD [9] take the entire image as input and directly extract global features, offering higher efficiency and real-time performance, albeit potentially at the cost of lower accuracy and precision. YOLOv5, the fifth iteration of the YOLO series, features an improved network architecture and optimized training strategies, leading to a substantial increase in detection precision. Recent advancements in YOLOv5 adaptations have significantly improved small object detection. YOLOv5 achieves a well-established balance between accuracy and speed, and it benefits from a large community that provides support and compatibility for various optimizations. Additionally, the architecture of YOLOv5 is relatively lightweight, making it easier to balance model complexity and performance, which is crucial for practical applications. While YOLOv8 and YOLOv10 have been released, YOLOv8 is an improvement based on YOLOv5, and YOLOv10 builds upon YOLOv8. YOLOv5 maintains a significant position in the field of object detection and continues to be widely used in real-world detection applications. Yang et al. introduced KPE-YOLOv5, which incorporates the scSE attention module to enhance the network’s focus on small object features, thereby increasing detection accuracy [10]. Zhang et al. proposed an Adaptive Slicing Approach (ASAHI), which optimizes processing speed and detection performance by altering the number of slices rather than their size, effectively reducing redundant computations [11]. Kim et al. developed ECAP-YOLO, which integrates ECA-Net into YOLOv5 to optimize attention on small objects and enhance feature representation [12]. Mahaur et al. improved the Spatial Pyramid Pooling (SPP) module and PANet structure by substituting dilated convolution for pooling operations to further strengthen feature expression [13]. Guo et al. introduced MSFT-YOLO, combining Transformer modules with a multi-scale feature fusion structure (BiFPN), addressing issues of background noise and poor defect detection in industrial settings [14]. Wang et al. presented FE-YOLOv5, which significantly enhances small object detection performance through innovative feature enhancement and spatial awareness technologies [15]. Dong et al. proposed a lightweight improvement model based on YOLOv5, combining C3Ghost and Ghost modules to reduce the model’s computational complexity, while introducing the CBAM attention mechanism to enhance feature extraction performance [16]. To address the issues of real-time performance and accuracy in multi-scale traffic sign detection, Wang et al. proposed an improved YOLOv5 network that significantly enhances the detection capability for multi-scale objects by incorporating AF-FPN and automatic data augmentation strategies [17]. Liu et al. developed a model based on an improved YOLOv5, integrating a bidirectional Feature Pyramid Network (BiFPN) and a SimAM attention module to enhance small object detection capabilities [18].

Although these studies have achieved certain successes in improving small object detection accuracy, current models still have limitations when handling complex scenarios such as low light, illumination, and cluttered backgrounds. This study introduces the following enhancements based on the YOLOv5 model:

1. Integrate the Convolutional Block Attention Module (CBAM) into the YOLOv5 model, that is, include the CBAM [19] attention mechanism into the C3 module of the backbone network to improve its feature representation capabilities, to improve the model’s capture ability for important parts of the image to increase the accuracy of detecting image fragments.
2. Replace the Spatial Pyramid Pooling-Fast (SPPF) module with the Receptive Field Block (RFB) module [20] to expand the receptive field of the original model and better and correct use of contextual information, which may improve the perception of objects of different sizes and shapes by the transformed model and may lead to an increase in the accuracy of detecting small objects.
3. Adapt the basic model by adding a new detection and identification layer on top of the existing network architecture, focused on identifying small objects and making full use of shallow features for more accurate localization of small targets in the image, with the aim of further increasing the accuracy and correctness of detecting small objects.

In addition, although the proposed improvements are based on YOLOv5, their modular design provides the potential for integration and optimization in future versions of YOLO. These improvements, when combined with the latest advancements in these versions, can further enhance the model’s performance across various application scenarios, particularly in small object detection.

## 2. Materials and Methods

**Figure 1:**
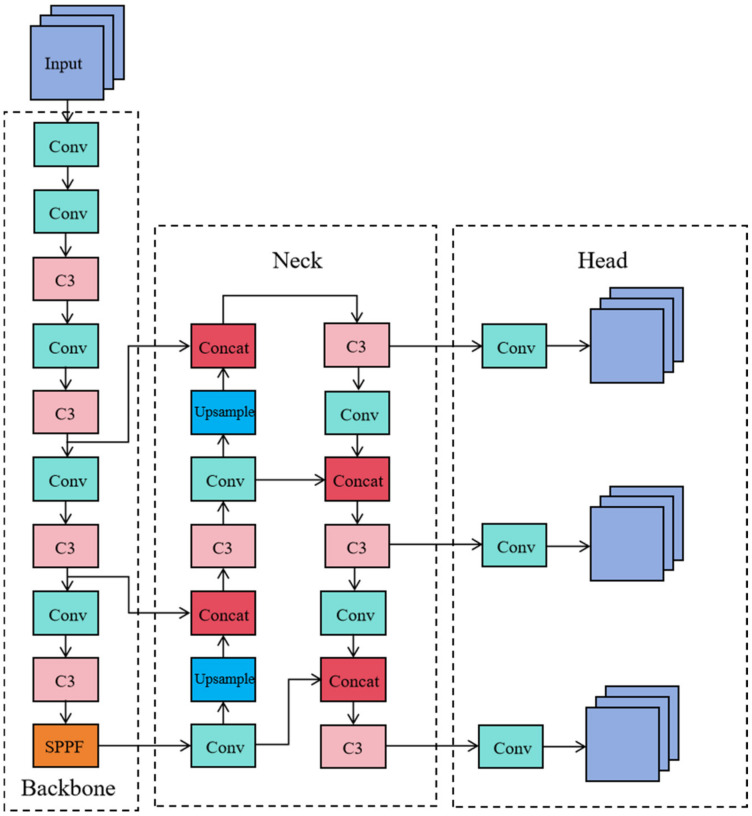
YOLOv5 network structure.

This research builds upon the YOLOv5 model. Figure 1 illustrates the network structure of YOLOv5, which is divided into three main components: the backbone network, the neck network, and the output section. During object detection, the input image is first resized to a standard dimension of 640 × 640 pixels. The backbone network then extracts features, generating feature maps of various sizes, which are passed to the neck network for feature fusion. The neck network integrates the structures of Feature Pyramid Network (FPN) [21] and Path Aggregation Network (PAN) [22], enabling the transfer of semantic information from deeper layers to shallower ones and the feedback of positional information from shallower to deeper layers. This process results in a feature pyramid that combines both semantic and location information [23]. Finally, the fused feature maps in the neck are processed by convolutional modules to output detections for objects at different scales—large, medium, and small.

**Figure 2:**
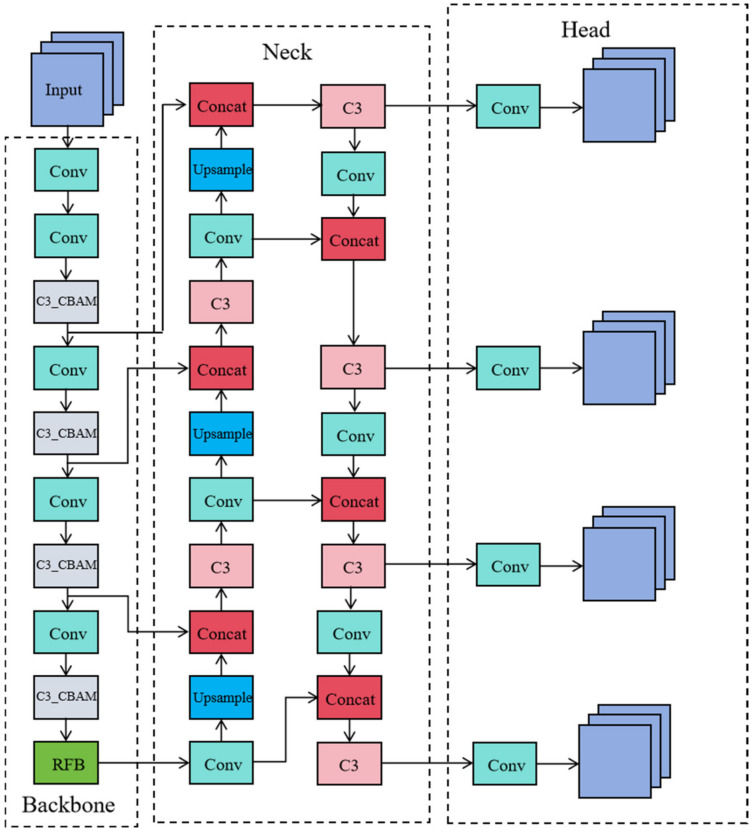
Improved YOLOv5 network structure.

To enhance the network’s feature extraction capabilities, the CBAM attention mechanism is integrated into the C3 module of YOLOv5’s backbone network, optimizing the distribution of feature weights. Additionally, the SPPF module in the backbone is replaced with an RFB module, which broadens the model’s receptive field and improves the integration of multi-scale features, thereby enhancing detection precision. Furthermore, a new detection layer specifically designed for small objects is added to the network structure, maximizing the use of shallow features. These modifications collectively enable the enhanced YOLOv5 to achieve higher detection accuracy. The updated network structure is illustrated in Figure 2.

### 2.1. C3_CBAM Module

The Convolutional Block Attention Module (CBAM) consists of two key components: the Channel Attention Module (CAM) and the Spatial Attention Module (SAM). CAM enhances the model’s ability to distinguish important features by adjusting the significance of different channels. In contrast, SAM improves the model’s capacity to capture positional information by extracting critical details from the spatial dimension.

**Figure 3:**
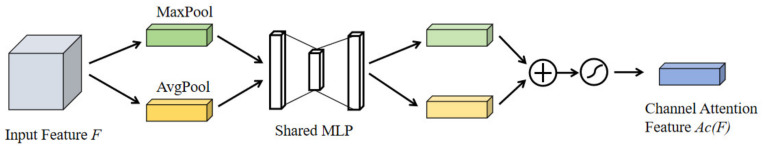
Schematic diagram of CAM principles.

Figure 3 is the principle of the Channel Attention Module (CAM). Initially, the input feature map *F* undergoes both max pooling and average pooling operations, yielding the max-pooled feature map $F_{\max}^{c}$ and the average-pooled feature map $F_{avg}^{c}$, respectively. These feature maps are then fed into a fully connected layer responsible for learning the attention weights for each channel. This process allows the model to adaptively recognize and emphasize the channels that are more important for the current task. Subsequently, the global maximum and average feature vectors are combined to form the final attention weight vector. A sigmoid activation function is employed to ensure that the channel attention weights are scaled between 0 and 1. These weights are then applied to each channel of the original feature map [24]. The resulting attention-weighted channel feature map, *Ac*(*F*), is produced by multiplying the derived attention weights with each channel of the original feature map, emphasizing channels that are beneficial for the current task while suppressing irrelevant ones. The processed channel feature map *Ac*(*F*) can be expressed as follows, where *σ* denotes the sigmoid activation function, and *W*_1_ and *W*_2_, respectively, represent the weight matrices of the first and second hidden layers in the fully connected layer.

(1) $$Ac(F)=\sigma (W_{2}(W_{1}(F_{\max}^{c}))+W_{2}(W_{1}(F_{avg}^{c})))$$

**Figure 4:**
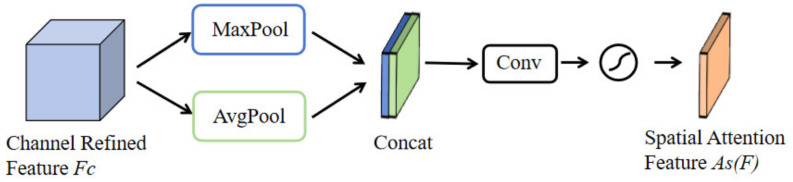
Schematic diagram of SAM principles.

Figure 4 is the operational principle of the Spatial Attention Module (SAM): Initially, the channel-optimized feature map *F_c_* undergoes max pooling and average pooling along the channel dimension, followed by concatenation of the two pooled feature maps. This forms a composite feature map that encapsulates contextual information at different scales. The composite feature map is then further processed through a convolutional layer to produce the spatial feature map *As*(*F*). Similar to the Channel Attention Module, the creation of the spatial feature map also utilizes a sigmoid activation function to ensure that attention weights are scaled between 0 and 1. This processing highlights significant areas within the image and reduces the impact of less important regions. The spatial feature map *As*(*F*) can be expressed as follows, with *σ* representing the sigmoid activation function and * denoting the convolution operation.

(2) $$As(F)=\sigma (*([MaxPool(F);AvgPool(F)]))=\sigma (*([F_{\max};F_{\mathrm{avg}}]))$$

**Figure 5:**
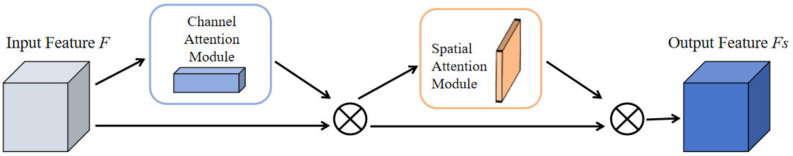
Schematic diagram of CBAM principles.

CBAM combines the output features from CAM and SAM by weighting them to produce the final attention-enhanced features. These features are then used as input for subsequent network layers, preserving key information while suppressing noise and irrelevant details. The operational principle of CBAM is illustrated in Figure 5 and can be mathematically represented as follows, with *F_c_* representing the feature map optimized by the Channel Attention Module, and *F_s_* representing the feature map optimized by the Spatial Attention Module.

(3) $$F_{c}=Ac\left(F\right)⊗F$$

(4) $$F_{s}=As\left(F_{c}\right)⊗F_{c}$$

**Figure 6:**
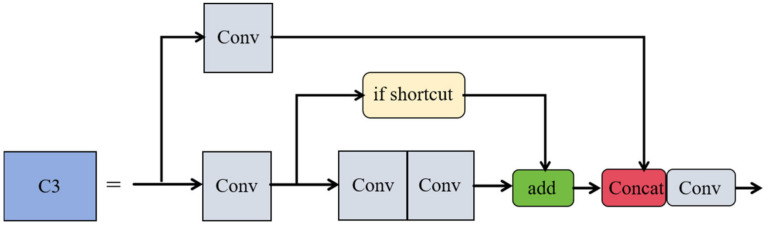
C3 module structure diagram.

To augment the feature extraction capabilities of the network, this study integrates the CBAM attention mechanism with the C3 module of the YOLOv5 backbone, forming the feature enhancement module C3_CBAM. The structure of the C3 module, as shown in Figure 6, consists of two residual blocks, each extracting rich features through multi-layer convolution. In this configuration, the Shortcut option is set to true by default, allowing the input feature map to be added to the output feature maps processed by two convolutional layers, thereby forming residual connections. Furthermore, the Add operation performs a simple pixel-level addition to increase the information content. The Concat operation, on the other hand, concatenates the output feature maps of the residual blocks with the feature maps from the skip connections along the channel dimension, thus creating new feature maps with richer content.

**Figure 7:**
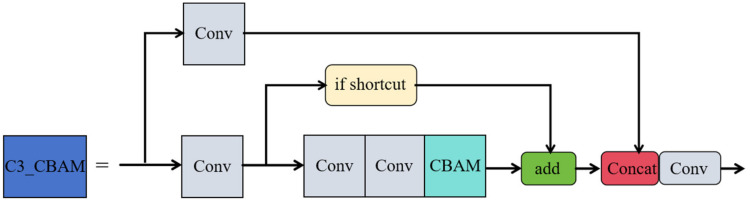
C3_CBAM structure diagram.

The CBAM attention mechanism is integrated into the residual blocks of the C3 module in YOLOv5, forming a new feature extraction module called C3_CBAM, as illustrated in Figure 7. The incorporation of CBAM is intended to improve the model’s ability to recognize features of small objects, particularly by enhancing the feature channels that are crucial for small object identification. This integration not only increases the model’s sensitivity to small object features but also sharpens its focus on critical spatial information while reducing the impact of irrelevant background details. As a result, this enhancement leads to more precise and reliable localization and identification of small objects.

### 2.2. RFB Module

**Figure 8:**
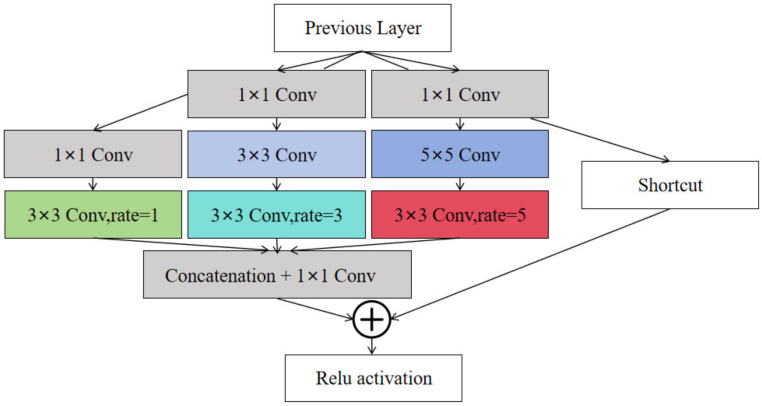
RFB module structure diagram.

The SPPF module in the YOLOv5 network structure helps to expand the model’s receptive field and gather global information, but it performs moderately in handling precise multi-scale object detection tasks. Inspired by the human visual receptive field, the design of the RFB module is apt for processing complex and varied visual information. Replacing the SPPF module with the RFB module further enlarges the model’s receptive field and enhances the acquisition of comprehensive contextual information. The structure of the RFB module, as shown in Figure 8, utilizes a combination of multi-branch convolution and dilated convolution. The features processed by convolution are concatenated and then fused through a 1 × 1 convolution, producing the final output feature map. By employing convolutional kernels of various sizes and dilated convolutions, the RFB module effectively captures multi-scale features, significantly boosting the model’s capability to detect objects with substantial differences in shape and size. The use of dilated convolution not only enlarges the receptive field without additional computational cost but also maintains the resolution of the feature map while improving the efficiency of processing large area contextual information. This design enhances the model’s discriminative power and robustness, making it more efficient and accurate in facing diverse detection tasks.

### 2.3. Adding a Dedicated Small Object Detection Layer

The YOLOv5 network incorporates an FPN and PAN structure, where the FPN is responsible for conveying deep semantic information to the shallower layers, and the PAN transfers shallow positional information to the deeper layers. This architecture effectively merges features from different levels, enhancing the network’s ability to detect objects. The network’s three output heads are designed to detect small-, medium-, and large-scale objects, respectively. However, as the resolution of feature maps decreases with network depth, fine details of small objects are often lost, which can negatively impact their accurate localization and identification. To address this issue, an additional layer dedicated to small object detection is introduced, processing higher resolution feature maps. This added layer enables the model to detect objects across various scales, enhancing its multi-scale detection capability and allowing it to handle targets of different sizes simultaneously. As a result, the network achieves more accurate recognition and localization in complex scenes. The input to this new small object detection layer is derived from shallower feature layers of the backbone network, and it outputs smaller-sized detection heads. This design captures more detail, further improving the performance of small object detection.

## 3. Experiments and Analysis

### 3.1. Experimental Data and Environment

**Figure 9:**
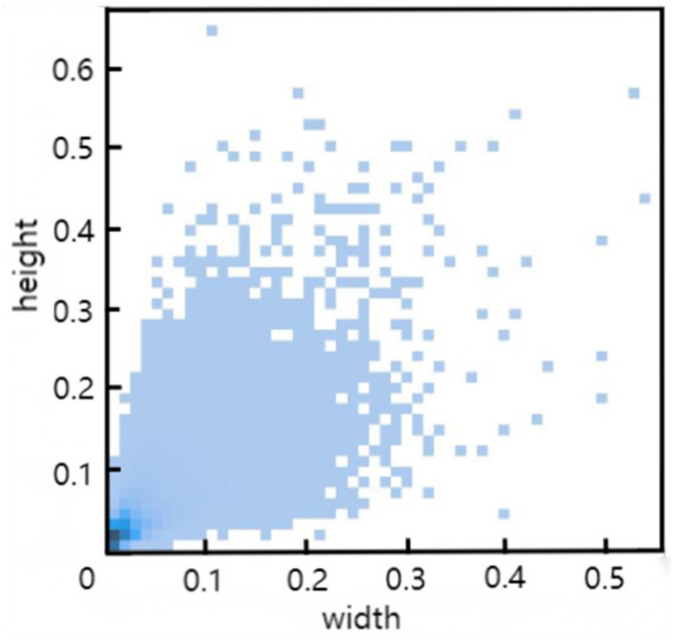
Distribution of all label sizes in the training set.

The dataset selected for the experiments in this study is VisDrone2019, which is shared by the AISKYEYE team at Tianjin University. It consists of 10,209 images, with 6471 images used for training, 3190 for testing, and 548 for validation. The dataset includes labels for ten categories: pedestrians, people, bicycles, cars, vans, trucks, tricycles, awning-tricycles, buses, and motorcycles. Figure 9 shows the distribution of all label sizes within the training set, with the *x*-axis representing label width and the *y*-axis representing label height. As observed, the labels are densely packed in the lower left corner, indicating a prevalence of small objects, making this dataset well-suited for this study.

The experiments were conducted on a server running Ubuntu 22.04 operating system, utilizing Python 3.10 as the programming language. The deep learning framework used was PyTorch 2.1.0, with CUDA 12.1 as the parallel computing platform. The hardware included an RTX 4090 GPU. All experiments were performed with consistent hyperparameters across tests. Specifically, the training was carried out for 150 epochs with a batch size of 16, an initial learning rate of 0.01, and an image resolution of 640 × 640 pixels.

### 3.2. Evaluation Metrics

The performance of the model in this experiment was evaluated using several metrics: precision (P), recall (R), mAP50, mAP50-95, the number of parameters, GFLOPs, and FPS. Precision (P) measures the accuracy of the model, defined as the ratio of correctly predicted positive observations to the total predicted as positive. Recall (R) assesses the model’s ability to capture all relevant cases, defined as the ratio of correctly identified positive cases to all actual positive cases. AP represents the area under the precision–recall (PR) curve, and the mean average precision (mAP) is calculated by averaging the AP across all categories. mAP50 refers to the mean precision at an Intersection over the Union (IoU) threshold of 0.5; it is the most used metric for measuring accuracy in object detection. It intuitively reflects the overall performance of the model in object detection, especially in small object detection scenarios. mAP50-95 refers to the mean precision across the IoU threshold ranging from 0.5 to 0.95, making it a more stringent evaluation metric. It provides a more comprehensive reflection of the model’s detection performance across different object sizes and scenarios, particularly for precise small object localization. The number of parameters indicates the total parameters used by the model. GFLOPs measure the billions of floating-point operations the processor can perform per second, evaluating the complexity and computational demand of the model or algorithm. A lower GFLOPs value indicates that the model demands fewer computational resources, making it suitable for resource-limited or real-time applications. Frames Per Second (FPS) represents the number of image frames processed per second, which directly reflects the model’s real-time capability and efficiency.

### 3.3. Ablation Study and Analysis of Algorithm Effectiveness

To evaluate the impact of the number of CBAMs introduced into the backbone network on model performance, we designed a set of experiments. By comparing the increase or decrease in the number of C3_CBAMs in the backbone, we tested the model’s performance in detecting small objects. The experimental results are shown in Table 1. This experiment consists of five groups, corresponding to no CBAMs being added to the C3 module and the incremental addition of CBAMs to the C3 module.

As shown in Table 1, with the increasing number of C3_CBAM modules in the backbone network, the model’s accuracy gradually improves compared to the original YOLOv5 model, while both the parameter count and model complexity decrease. Notably, the approach that integrates the CBAM attention mechanism into all four C3 modules achieves the most significant improvement, with an increase of 0.6% in mAP50. The addition of C3_CBAM modules has minimal impact on the parameter count and model complexity, regardless of the number of modules introduced. This demonstrates that as the number of C3_CBAM modules increases, the attention mechanism is applied across multiple network layers, progressively enhancing the model’s focus on key features, thereby offering a clear advantage in small object detection.

To demonstrate the effectiveness of the three approaches utilized, ablation studies were conducted under identical conditions to investigate the impact of each method on algorithm accuracy and computational complexity. The results of the ablation experiments are presented in Table 1. In the table, ‘A’ represents the use of C3_CBAM, ‘B’ denotes the addition of a small object detection layer, and ‘C’ indicates the replacement of the SPPF module with the RFB module.

As shown in Table 2, the use of each improvement method independently resulted in enhanced model accuracy, precision, and recall compared to the baseline YOLOv5s model. Notably, the addition of a small object detection layer showed the most significant improvement, with a 5.2% increase in mAP50, underscoring the importance of accurate positional information for detecting small objects. This method effectively utilizes shallow positional information to enhance the localization accuracy of small objects. When combining multiple methods, the highest detection accuracy was achieved using C3_CBAM, the small object detection layer, and the RFB module simultaneously. Compared to the baseline YOLOv5s model, this combination led to a 5.4% increase in mAP50, a 3.6% increase in mAP50-95, a 5.1% increase in precision, and a 4.2% increase in recall, with the number of parameters increasing from 7.05 M to 7.87 M. These results indicate that a slight increase in the number of parameters effectively enhances detection accuracy.

**Figure 10:**
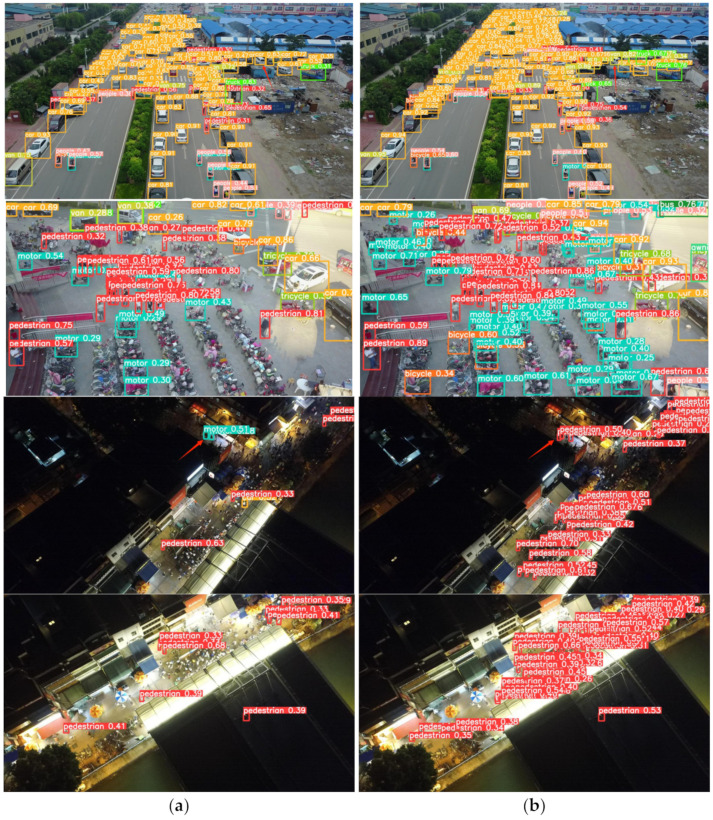
Comparison of test results. (**a**) YOLOv5 baseline. (**b**) CRL-YOLOv5.

Figure 10 presents the comparative test results of baseline YOLOv5 and enhanced CRL-YOLOv5 in various scenes. The test images were selected from the VisDrone2019 test set and included scenes such as roads, shopping malls, night-time, and illuminated conditions. These scenes contain a rich and dense population of small objects, and these objects represent most of the object types in the dataset, meeting the requirements of the detection task. Figure 10a displays the detection results of the baseline YOLOv5, while Figure 10b shows the results from the enhanced CRL-YOLOv5. It is evident that YOLOv5 missed many targets in these scenarios, whereas the improved algorithm successfully detected most of the small objects, significantly reducing the number of misses. Additionally, in the first image, YOLOv5 misclassified the van on the right as a car, and in the third image, it incorrectly identified a pedestrian as a motor. These issues did not occur with the improved algorithm, which effectively reduced the false detection rate. In densely populated areas across all four images, YOLOv5’s detection performance was consistently inferior to that of the improved CRL-YOLOv5. Furthermore, under night-time and low-light conditions, YOLOv5’s detection results were noticeably worse. In conclusion, the upgraded CRL-YOLOv5 algorithm exhibited superior performance in detecting small objects, significantly reducing misses and false detections, making it suitable for practical detection scenarios where targets are densely packed, or lighting is insufficient.

### 3.4. Comparative Experiments

Finally, the enhanced CRL-YOLOv5 algorithm was compared with major algorithms in the field of object detection, using detection accuracy and speed as evaluation metrics. The results of these comparative experiments are shown in Table 3. The table indicates that, compared to previous iterations of YOLOv5 and other typical models, our algorithm achieves higher accuracy, with an mAP50 that is 8.5% higher than Faster-RCNN and 6.1% higher than YOLOv4. Although the FPS is 27, which is a decrease compared to YOLOv4 and YOLOv5, it still maintains real-time detection capability. Compared to TPH-YOLOv5, a model specifically designed for small object detection, our model shows a 1.9% higher mAP50 and also exhibits improved FPS. Furthermore, it also outperforms the latest models in the YOLO series, with an mAP50 that is 0.3% higher than YOLOv8. Overall, the results demonstrate that the CRL-YOLOv5 algorithm possesses significant advantages in handling small object detection tasks.

## 4. Conclusions

Addressing the challenges of low accuracy, missed detections, and false positives in small object detection in images, this paper proposes an improved algorithm, CRL-YOLOv5. Firstly, the algorithm integrates the CBAM within the residual structures of the C3 module in the backbone network to redistribute feature weights, enhancing the localization and detection capabilities for small objects. Secondly, a new small object detection layer is added to augment multi-scale feature fusion, aiding in the precise localization of small objects. Lastly, the substitution of the SPPF module with an RFB module expands the model’s receptive field, improving the network’s ability to capture features of small objects. Experiments conducted using the VisDrone2019 dataset demonstrate that the enhanced model achieves improved detection accuracy, exhibits effective performance on small objects, and is suitable for everyday small object detection scenarios. The proposed method also holds potential for application in radiographic and tomographic image processing in medical imaging and non-destructive industrial testing. CRL-YOLOv5 has demonstrated significant improvements in detection accuracy, and future research could explore lightweight models to reduce computational resource requirements.

## Figures and Tables

**Table 1:** C3_CBAM increase/decrease experiment.

C3_CBAM Quantity	mAP50/%	mAP50-95/%	Params/M	GFLOPs
0	33.8	18.7	7.05	16.0
1	33.9	18.8	6.72	15.0
2	34.1	18.8	6.72	15.0
3	34.2	18.9	6.72	15.0
4	34.4	18.9	6.73	15.0

**Table 2:** Results of ablation experiments.

YOLOv5s	A	B	C	mAP50/%	mAP50-95/%	P/%	R/%	Params/M	GFLOPs
√				33.8	18.7	45.4	34.5	7.05	16.0
√	√			34.4	19.0	48.8	34.6	6.73	15.0
√		√		39.0	22.1	49.8	39.1	7.19	18.9
√			√	34.4	19.1	47.6	34.9	7.71	16.6
√	√	√		39.0	22.1	50.3	38.5	7.21	19.0
√	√	√	√	39.2	22.3	50.5	38.7	7.87	19.5

**Table 3:** Comparison experiment results.

Algorithm	mAP50/%	mAP50-95/%	FPS
Faster-RCNN	30.7	16.1	17
YOLOv4 [25]	33.1	18.1	36
YOLOv5s	33.8	18.7	47
TPH-YOLOv5 [26]	37.3	20.2	15
YOLOX [27]	35.2	19.4	77
YOLOv7 [28]	36.7	20.8	89
YOLOv8	38.9	22.1	86
CRL-YOLOv5	39.2	22.3	27

## Data Availability

The original data presented in this study are openly available at https://github.com/VisDrone/VisDrone-Dataset (accessed on 24 September 2023).
